# Childhood Gender Nonconformity and Recalled Perceived Parental and Peer Acceptance Thereof, Internalized Homophobia, and Psychological Well-Being Outcomes in Heterosexual and Gay Men from Poland

**DOI:** 10.1007/s10508-021-02245-9

**Published:** 2022-06-02

**Authors:** Monika Folkierska-Żukowska, Qazi Rahman, Wojciech Ł. Dragan

**Affiliations:** 1 Faculty of Psychology, University of Warsaw, Ul. Stawki 5/7, 00-183, Warsaw, Poland; 2 Department of Psychology, Institute of Psychiatry, Psychology, & Neuroscience, King’s College London, London, UK

**Keywords:** Minority stress, Childhood gender nonconformity, Depression, Social anxiety, Internalized homophobia, Sexual orientation

## Abstract

The link between gender nonconformity and psychopathology may be due in part to negative childhood experiences resulting from other people’s reactions to gender nonconformity. The aim of this study was to test whether recalled perceived levels of parental and peer acceptance of childhood gender nonconforming behaviors and play mediate the relationship of childhood gender nonconformity with depression and social anxiety in adulthood. We also tested whether this relationship was moderated by sexual orientation and, among gay men, whether internalized homophobia was an additional mediator. All variables were measured in a large sample of male participants using self-report (*n* = 449 gay men, age: M = 27.8 years, SD = 6.69; and *n* = 296 heterosexual men, age: M = 27.4 years, SD = 6.57) in Poland. Gay men reported more childhood gender nonconformity than heterosexual men. The relationship between gender nonconformity and depressive symptoms as well as social anxiety symptoms was significant in both gay and heterosexual men. Among gay men, this relationship was partially mediated by peer but not parental acceptance of the measured aspects of gender nonconformity and internalized homophobia. Among heterosexual men, recalled perceived parental acceptance of gender nonconformity partially mediated the relationship between gender nonconformity and depressive and social anxiety symptoms. Our findings were partially in line with those found in Western European and North American samples. Although the two groups differed in their recalled perceived gender nonconformity, they did not differ in their depression or social anxiety scores. Nevertheless, childhood gender nonconformity may be an indirect risk associated with mental health symptoms, irrespective of sexual orientation. Its higher prevalence among nonheterosexual individuals makes it a particular risk for this group.

**Supplementary Information:**

The online version contains supplementary material available at 10.1007/s10508-021-02245-9.

## Introduction

Research suggests that nonheterosexual men and women have higher rates of common mental health problems (including depression and anxiety) compared to heterosexual people (King et al., 2008; Plöderl & Tremblay, 2015). One hypothesis is that these elevated rates of certain mental health outcomes are due to minority stress: experiences of discrimination and social stigma due to being a sexual minority which cascade into poorer mental health (Meyer, 1995, 2003). Minority stress theory and its various modifications propose a range of possible psychosocial mediators of the relationship between discrimination or stigma and mental health, including factors such as internalized homophobia (Hatzenbuehler, 2009; Meyer, 2003). Exposure to minority stressors may occur at various stages of one’s life. For example, they may appear quite early in childhood when behavioral markers of later nonheterosexuality, most notably gender nonconformity, are already present (D'Augelli et al., 2008; Hockenberry & Billingham, 1987; Zucker et al., 2006).

Retrospective (Rieger et al., 2008) and prospective (Li et al., 2017; Xu et al., 2020) studies have robustly demonstrated that gender nonconformity is a strong developmental correlate of nonheterosexuality. The association can be observed early in childhood and persists into adulthood (Li et al., 2017; Lippa, 2005; Rieger et al., 2008, 2010). The effect size is large for recalled childhood gender nonconformity (CGN; Cohen’s *d*’s are about 1.31 and 0.96 for differences between heterosexual men and women, and gay men and lesbian women, respectively; Bailey & Zucker, 1995) and moderate to large for adult gender nonconformity (Lippa, 2005; Rieger et al., 2010). Other people also perceive gay men and lesbian women as more gender nonconforming than heterosexual people based on their gestures, appearances, and speech patterns (e.g., Johnson et al., 2007; Rieger et al., 2010; Valentova & Havlíček, 2013). These relationships also appear moderate to large in effect size (Rieger et al., 2008, 2010).

In addition to the known association between sexual orientation and mental disorders, recalled CGN has been associated with poorer mental health outcomes, including symptoms of depression (Roberts et al., 2013), general psychological distress (Alanko et al., 2009), and social anxiety (Feinstein et al., 2012). These associations are complex and found both independently of sexual orientation and sometimes in interaction with it. For example, one study reported that CGN was associated with depression in men independently of their sexual orientation (Roberts et al., 2013). Another study reported that gender nonconformity in childhood and adolescence was negatively associated with psychological well-being, but that neither sex nor sexual orientation was significant predictors of well-being scores (Rieger & Savin-Williams, 2012). A further study reported that gender nonconformity and same-sex attraction were associated with psychopathology in men and women (Alanko et al., 2009).

The association between gender nonconformity and psychopathology may be due to experiences of stigmatization during development. That is, negative childhood experiences resulting from other people’s reactions to gender nonconformity may cascade into poorer mental health. First, as reported by D'Augelli et al. (2008), nonheterosexual youths report feeling different by about 8 years of age and that their “difference” was pointed out to them by others at a similar age. Second, CGN has been associated with experiencing bullying (Friedman et al., 2006). It has also been linked with negative parent–child relationships (Roberts et al., 2012a, 2013), and abuse (Sandfort et al., 2007), as well as rejection by peers and family members (Fagot, 1985; Langlois & Downs, 1980; Smith & Leaper, 2006). Negative parental reactions to CGN can be a result of perceiving CGN as a sign of future nonheterosexuality (D’Augelli et al., 2006; Sanborn-Overby & Powlishta, 2020). These lines of evidence suggest that the association between CGN and poor mental health in adulthood is mediated by factors related to one’s environment and reactions to CGN.

Negative consequences of CGN seem to be worse for boys than for girls (Coyle et al., 2016; Fagot, 1985; Kwan et al., 2020; Langlois & Downs, 1980; Roberts et al., 2013; Sanborn-Overby & Powlishta, 2020; Young & Sweeting, 2004). Some reports found associations between CGN and adult mental health for men but not for women (Lippa, 2008; Petterson et al., 2017) or found that these associations were stronger for men than for women (Feinstein et al., 2012; Roberts et al., 2013; cf. Rieger & Savin-Williams, 2012). These sex differences may be due to violations of cultural gender norms and greater social toleration of gender nonconformity among girls, as masculine behaviors in women are generally perceived as more positive (e.g., Coyle et al., 2016; D’Augelli et al., 2006; Kane, 2006).

In terms of mental health outcomes, CGN has been found to be associated with higher levels of separation anxiety (Santarossa et al., 2019) as well as behavioral and emotional challenges in children aged 6–12, which was stronger if the primary caregivers endorsed gender stereotypes and if the child had poor peer relations (MacMullin et al., 2021). Alanko et al. (2009) found that levels of recalled CGN correlated with negative recalled ratings of parent–child relationships (corresponding to the authoritarian parenting style), and that both gender nonconformity and negative parent–child relationships predicted adult depression and anxiety symptoms. Friedman et al. (2006) found that reported bullying mediated the relationship between recalled CGN and suicidality in young adult gay men. D’Augelli et al. (2006) found that recalled perceived peer and parental responses to CGN in gay, lesbian, and bisexual individuals were related to current mental health and that individuals who reported gender nonconformity also reported having experienced more physical violence in their lifetime.

Apart from parental and peer reactions to gender nonconformity, including rejection (Friedman et al., 2006; Ryan et al., 2009), other psychosocial factors that mediate the association between CGN and poor mental health may include internalized homophobia (Meyer, 2003; Newcomb & Mustanski, 2010). Internalized homophobia has also been robustly linked to poorer mental health among homosexual individuals (particularly with internalizing forms of psychopathology such as depression and anxiety; Newcomb & Mustanski, 2010). Internalized homophobia is a construct applicable to sexual minorities: It refers to a negative perception of one’s own sexual orientation due to the internalization of homophobic social attitudes (Herek et al., 1998). Minority stress models of sexual minority health disparities often conceptualize internalized homophobia as an indirect or proximal risk factor that mediates between distal or direct risk factors (e.g., homophobic abuse from others) and mental health outcomes (Hatzenbuehler, 2009; Meyer, 2003). Factors such as gender nonconformity may be hypothesized to be antecedent or moderator risk factors, although they tend to be neglected in standard minority stress frameworks (cf. Timmins et al., 2020). It is possible that internalized homophobia is made worse by experiences of childhood victimization (including negative reactions from parents and peers) as a consequence of being gender nonconforming. One study reported that direct homophobic victimization and internalized homophobia partially mediated the relationship between adult (not childhood) gender nonconformity and psychological distress (using the Brief Symptom Inventory; Van Beusekom et al., 2018). Gender nonconformity was associated with greater psychological distress via direct homophobic victimization experiences but also with lower psychological distress due to reduced levels of internalized homophobia. However, the complex associations between CGN, recalled parental and peer reactions to gender nonconformity, internalized homophobia, and mental health outcomes have not been studied.

In the current study, we decided to focus on depression and social anxiety for several reasons. Depression is one of the main mental health burdens across the world in general and specifically for LGBT populations (e.g., see King et al., 2008). Social anxiety is the most common form of anxiety disorder, with a lifetime rate of about 14%—much higher than other anxiety disorders in the population (e.g., Kessler et al., 1994). Importantly, social anxiety may be more prevalent or important for LGBT people because of fear of rejection by others, which is part of the minority stress model. Gay men in particular may expect rejection by heterosexual peers, co-workers, or family, and this may translate into symptoms of social anxiety (e.g., Hart & Heimberg, 2001; Meyer, 2003; Pachankis & Goldfried, 2006). Social anxiety might thus relate to both fear of rejection and also internalized homophobia. (Feeling uncomfortable with one's own identity might understandably also translate into social anxiety.)

In terms of sexual orientation, we decided to focus on exclusively homosexual men. We decided against including bisexual individuals in the study for several reasons. While there is some evidence to suggest a level of gender nonconformity also in bisexual individuals, they are not as gender nonconforming: Some reports suggest that their level of gender nonconformity is intermediate between heterosexual and homosexual individuals (e.g., Rieger et al, 2020). Moreover, it is likely that mental health risk among bisexuals might be unique to bisexual people, and while it may also be related to their gender nonconformity, this relationship is probably less strong than in exclusively gay/lesbian people. Other psychosocial risks unique to their sexual orientation may be at play here: For instance, they often face prejudice from not only the heterosexual majority, but also from lesbians and gay men (see, e.g., Mereish et al, 2017). There is growing evidence from population-level and community health and surveillance surveys that bisexual people are even more prone to common mental health problems than lesbian women and gay men (Chan et al., 2020; Colledge et al., 2015; Pitman et al., 2021; Semlyen et al., 2016).

The aim of the present study was to test the aforementioned complex associations in a survey study of Polish heterosexual and gay men. A Polish sample is interesting to study, since the country scores highly on measures of homophobic legal and policy practices within the European Union (ILGA-Europe, n.d.) and in social attitudes (Chojnicka, 2015). We argue that CGN is an antecedent risk for negative reactions from parents and peers (in response to the gender nonconformity) and that the relationship between CGN and mental health outcomes is partially mediated by these negative reactions and, subsequently, by internalized homophobia. Based on the existing literature, these patterns will be stronger in males, and so we focus on men in this study. Firstly, we predicted that gay men would show higher recalled CGN, depression, and social anxiety scores compared to heterosexual men. Secondly, we hypothesized that greater CGN would correlate with lower recalled perceived parental and peer acceptance of CGN, independently of sexual orientation, as suggested by previous literature (Alanko et al., 2009; D’Augelli et al., 2006; Friedman et al., 2006; Roberts et al., 2012b, 2013). We also hypothesized that recalled perceived levels of parental and peer acceptance of CGN would mediate the relationship between recalled CGN and depression and social anxiety symptoms. This pattern of associations was predicted to be stronger in gay men than in heterosexual men. That is, sexual orientation would act as a moderator of these relationships. Furthermore, it was hypothesized that, in the case of gay men, internalized homophobia would act as an additional mediator of this relationship.

## Method

### Participants

Participants were recruited in Poland via an online survey completed as a part of a larger project concerning biological and psychosocial correlates of male sexual orientation (Folkierska-Żukowska et al., 2020). The recruitment questionnaire was distributed through Facebook (paid adverts and organic traffic), leaflets, posters, and by word of mouth. The adverts were targeted at individuals living in several major Polish cities—Warsaw, the Tricity (metropolitan area of Gdańsk, Gdynia, and Sopot), Poznań, Kraków, and Wrocław—and sought heterosexual and gay men aged 18–45 who wanted to take part in a study about sexual orientation. Respondents were invited to read more information about the study (in Polish; http://icgz.uw.edu.pl/projekt-orientacja/) and informed that those who complete the survey may qualify for a face-to-face meeting (carried out in one of the aforementioned cities), for which they would be remunerated. This was completed by 3515 individuals between December 2016 and January 2020. Of these, 2285 fulfilled the inclusion criteria (being a predominantly heterosexual or predominantly homosexual cis-gendered man and aged 18–45 years) and were invited to the laboratory. A total of 945 men accepted the invitation, attended the laboratory, and completed all the measures (515 gay men and 430 heterosexual men). All participants who attended the laboratory received remuneration for participating (36 PLN or approximately 10 USD). The final sample consisted of those who answered the questions on the measure about recalled perceived parental and peer acceptance of CGN (see Measures section) and was 794 individuals (342 predominantly heterosexual men and 452 predominantly homosexual men). Demographic differences are shown in Table 1.

**Table 1:** Means and SDs for variables of interest by group (after excluding participants who did not respond to questions regarding acceptance of their CGN)

	Gay men (*n* = 452)		Heterosexual men (*n* = 342)	
	M	SD	M	SD
Age (years)	27.81	6.69	27.42	6.57
Education (years)	16.21	3.26	16.68	3.04
Gender Nonconformity	3.59	0.50	4.05	0.34
Parents' acceptance	62.23	23.62	66.33	23.44
Peers' acceptance	45.11	21.24	41.84	22.03
Depression symptoms	0.93	0.88	0.88	0.78
Social anxiety symptoms	1.41	0.89	1.34	0.82
Internalized homophobia	1.68	0.80	-	-

For recalled childhood gender nonconformity, the scale ranges from 1 to 5, where 1 corresponds to extremely feminine behavior (extreme gender nonconformity) and 5 to extremely masculine behavior (extreme gender conformity). For recalled parental and peer acceptance scales, the range is from 1 to 100, where 0 indicated no acceptance at all, 50 indicated indifference, and 100 indicated full acceptance. For depression symptoms and social anxiety scales, the range is from 1 to 4 where higher score indicates higher frequency of symptoms. For the internalized homophobia scale, the score can range between 1 and 5 where higher scores indicate higher levels of internalized homophobia

The online survey consisted of demographic questions, questions about sexual orientation, recalled childhood gender nonconformity, and recalled perceived parental and peer acceptance of CGN. Questions about internalized homophobia, depression, and social anxiety were answered by the participants during the meeting. The scales were completed on a computer, and the researcher would leave the room when a participant was doing this but would be available in case any questions arose. The order of the scales was randomized both in the online survey and in the one completed during the meeting. Other scales were also included in the survey that were not analyzed in this study. Participants gave informed consent before each stage of the study and were informed that some of the questions would be of a personal nature.

### Measures

#### Demographic Questions

Participants were asked about their birth year, the size of their area of residence (they selected from: “Village,” “Town of up to 50 000 residents,” “Town of up to 150 000 residents,” “City of up to 500 000 residents,” “City of over 500 000 residents”), the total number of years of their education, and the highest level of education completed (“Primary incomplete,” “Primary complete,” “Vocational,” “Secondary,” “Secondary vocational,” “Postsecondary,” “Higher”).

#### Sexual Orientation

Sexual orientation was assessed via a Polish adaptation (Wierzba et al., 2015) of the Sell Assessment of Sexual Orientation (Sell, 1996). This tool measures three dimensions of sexual orientation: sexual attractions, sexual contact, and sexual identity. Homosexuality and heterosexuality are measured separately. For the purposes of this study, we defined predominantly heterosexual men as those who had been sexually attracted to either 0 or 1 men in the past year; who had never been attracted to a man or were attracted to a man less than once per month; whose attraction to a man in the past year ranged from “*not at all*” to “*mildly*”; who had been sexually attracted to at least one woman in the past year; who were sexually attracted to a woman at least once a month; and who identified as “*not at all homosexual*” or “*slightly homosexual*” and as either “*very heterosexual*” or “*extremely heterosexual.*” Criteria for predominantly homosexual men were, mutatis mutandis*,* identical. Questions about sexual contact were not included in our criteria, as they have the limitation of being potentially constrained by social censure of same-sex sexuality. See Supplementary Material for analysis of correlations between the components of the scale. Only cis-gendered men (i.e., men who reported being assigned male at birth and currently identified as male) took part.

#### Recalled Childhood Gender Nonconformity

This was measured using questions 1–15 and 18–21 from a Polish adaptation of the Recalled Childhood Gender Identity/Gender Role Questionnaire (corresponding to Factor 1 of the scale, concerning gender conformity; Zucker et al., 2006). The scale was adapted to Polish by the authors of the current study for the aforementioned project, in collaboration with the scale’s author, using a standard translation and back-translation procedure. The questions concerned recollections regarding the following before the age of 12: perceived masculinity/femininity; preferred toys and games; admired/imitated characters from TV/movies; roles taken in pretend play; dress-up play; enjoyment of “feminine” clothing; a reputation as a “sissy” or “tomboy”; contentment as a boy or girl; perceived masculinity/femininity of appearance; cross-sex desires; playing with cosmetics and jewelry; the sexes of peers with whom one played sports; the genders of favorite playmates and best friend; activity levels; and resentment felt toward same-sex siblings. A mean score was calculated for each participant (absolute range was 1–5) where 1 corresponds to extremely feminine behavior (extreme gender nonconformity) and 5 to extremely masculine behavior (extreme gender conformity). The response “*I did not do this/feel this way*” to an item was not scored and was not included in the mean. Since the questioning of traditional gender roles has intensified in recent years, for clarity, we modified the instructions on the questionnaire by adding “In the case of the words ‘feminine’ and ‘masculine,’ we refer to their stereotypical definitions functioning in society.” Cronbach’s alpha was 0.93.

#### Recalled Perceived Parental and Peer Acceptance of Gender Nonconformity

The scale used to measure this variable was developed by the authors, and the English translation of the full scale can be found in Supplementary Material. We assessed recalled perceived acceptance of childhood gender nonconforming behaviors and gender nonconforming play (assessed for parents and peers separately) using a slider on a scale from 1 to 100, where 0 indicated no acceptance at all, 50 indicated indifference, and 100 indicated full acceptance. The participants were told that “Some toys and ways of playing are traditionally considered more boyish (e.g., cars, playing war), or more girly (e.g., dolls, playing house),” and that “Some behaviors not associated with play are traditionally considered more boyish (e.g., aggression), or more girly (e.g., crying),” and were then asked to assess the reactions they remember receiving before 12 years of age from parents and peers when they engaged in: (1) types of play that are considered more girly and (2) behaviors that are considered more girly. Participants had the option to indicate that questions do not apply to them as they never behaved or played this way or to indicate “*I don’t remember.*” Mean scores for recalled perceived (1) parental and (2) peer acceptance of gender nonconforming play and gender nonconforming behaviors were calculated. Scores ranged from 0 to 100, with higher scores indicating greater recalled acceptance of CGN. Participants who indicated that the questions do not apply to them and who remembered neither parental nor peer acceptance for either behaviors or play were excluded from analyses (*n* = 117). Cronbach’s alpha for recalled perceived parental acceptance scale in the current sample was 0.75 (0.75 in gay and 0.74 in heterosexual men), and for recalled perceived peer acceptance, it was 0.59 (0.64 in gay and 0.52 in heterosexual men).

#### Internalized Homophobia

For gay men only, this was measured using the Polish adaptation (Górska et al., 2017) of the Internalized Homophobia Scale—Revised (Herek et al., 2009). This 5-item scale includes items such as “I have tried to stop being attracted to men in general” and was assessed on a 5-level Likert-like scale, with answers ranging from “I strongly disagree” to “I strongly agree.” Higher mean scores indicate higher levels of internalized homophobia (ranging from 1 to 5). Cronbach’s alpha was 0.75.

#### Depression and Social Anxiety

Depressive and social anxiety symptoms were measured using a Polish version of two subscales from the Symptom Checklist-27-Plus (Hardt, 2008; Kuncewicz et al., 2014). The subscales included five items each, concerning currently experienced symptoms of depression (e.g., feelings of hopelessness and loss of joy) and social anxiety (e.g., fear of embarrassment, feeling insecure when being looked at). Participants were asked to rate the frequency of occurrence of each symptom on a 5-level scale ranging from “*never*” to “*very often.*” Cronbach’s alpha for the depression subscale was 0.89 and for the social anxiety subscale was 0.84. Scores for both subscales were calculated as a mean and ranged between 0 and 4.

### Statistical Analysis

Independent samples *t* tests were used for group comparisons of continuous demographic variables, recalled CGN, recalled perceived parental and peer acceptance of CGN, depression, social anxiety, and internalized homophobia. Pearson’s correlations were used in correlational analyses.

Bootstrap tests were used to test the indirect associations, as this method is superior to the traditional Sobel’s test (Preacher & Hayes, 2004, 2008; Zhao et al., 2010). Moderated mediation analysis with 5000 bootstrapped resamples was performed using PROCESS (Hayes, 2013), with the relationship between CGN and mental health scores (depression and social anxiety symptoms) being tested for mediation by recalled perceived parental and peer acceptance levels, and sexual orientation acting as a moderator. For gay men only, a further mediation analysis with two mediators acting in serial was conducted. In these models, recalled perceived parental and peer acceptance and internalized homophobia were tested as mediators of the relationship between childhood gender conformity and depression and social anxiety scores. Age was included as covariate in all models. All variables were *z*-standardized before being entered into the model. The critical alpha for all statistical analyses was set to *p* = 0.05.

## Results

### Participant Characteristics

The groups did not differ significantly in terms of age, years of education (Table 1), highest completed level of education, or area of residence (Table 2)*.* As expected, gay men reported significantly less gender conformity than heterosexual men, *t*(784.39) = − 15.22, *p* < 0.001, *d* = -1.04. There were no significant differences between heterosexual and gay men on current depression, *t*(772.49) = 0.85, *p* = 0.395, *d* = 0.06, or social anxiety symptom subscale scores, *t*(762.64) = 1.24, *p* = 0.214, *d* = 0.09. Reported levels of recalled perceived parental acceptance of CGN differed significantly between gay and heterosexual men, *t*(792) = -2.53, *p* = 0.012, *d* = − 0.18. Reported levels of recalled perceived peer acceptance of CGN differed significantly between gay and heterosexual men, *t*(792) = 2.13, *p* = 0.034, *d* = 0.15 (Table 1).

**Table 2:** Frequencies of highest level of completed education and type of area of residence for the two final groups (after excluding participants who did not respond to questions regarding acceptance of their CGN)

	Gay men (*n* = 452)		Heterosexual men (*n* = 342)	
	Frequency	Percent	Frequency	Percent
Highest completed level of education				
Primary incomplete	0	0	0	0
Primary complete	6	1.33	8	2.33
Vocational	2	0.44	1	0.29
Secondary	132	29.2	119	34.7
Secondary vocational	18	3.98	13	3.79
Postsecondary	19	4.20	25	7.29
Higher	275	60.8	177	51.6
Type of area of residence				
Village	19	4.20	19	5.5
Town of up to 50 000 inhabitants	26	5.75	24	7.00
Town of up to 150 000 inhabitants	9	1.99	7	2.04
City of up to 500 000 inhabitants	58	12.8	37	10.8
City of over 500 000 inhabitants	340	75.2	256	74.6

### Moderated Mediation Analyses

#### Recalled Perceived Parental Acceptance and Depressive Symptoms

**Fig. 1:**
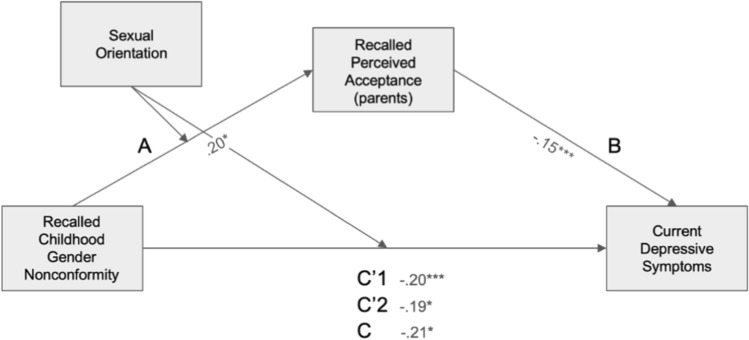
Mediated moderation model of the association between recalled childhood gender nonconformity and current depression scores mediated by recalled perceived parental acceptance levels and moderated by sexual orientation. Path coefficients are marked on the figure. Path statistics: A: *t*(789) = 2.53, *p* = .012; B: *t*(788) = -4.29, *p* < .001; C: *t*(789) = -5.42, *p* < .001; C’1 (gay): *t*(788) = -4.37, *p* < .001; C’2 (heterosexual): *t*(788) = -2.47, *p* = .014. *significant at *p* < 0.05, ** significant at *p* < 0.01, *** significant at *p* < 0.001. Note that a higher score on the gender nonconformity scale corresponds to higher masculinity and therefore to lower childhood gender nonconformity

The model with coefficients and statistics for all the indirect and direct paths is presented in Fig. 1. In the final model, *R*^2^= 0.096, *F*(5, 788) = 16.7, *p* < 0.001. The direct association between CGN and depression scores for heterosexual men was significant (β = − 0.19, *p* = 0.014, 95% CI [− 0.33, − 0.038]) as it was for gay men (β = −0.20, *p* < 0.001, 95% CI [− 0.28, − 0.11]). The indirect association through recalled perceived parental acceptance of CGN was also significant for heterosexual men (β = − 0.029, 95% CI [− 0.058, 0.006]), but not significant for gay men (β = − 0.013, 95% CI [− 0.031, 0.001]). However, the index of moderated mediation was nonsignificant, indicating that conditional indirect associations did not differ significantly between the two groups (β = 0.016, 95% CI [-0.011, ULCI = 0.045]).

#### Recalled Perceived Peer Acceptance and Depressive Symptoms

**Fig. 2:**
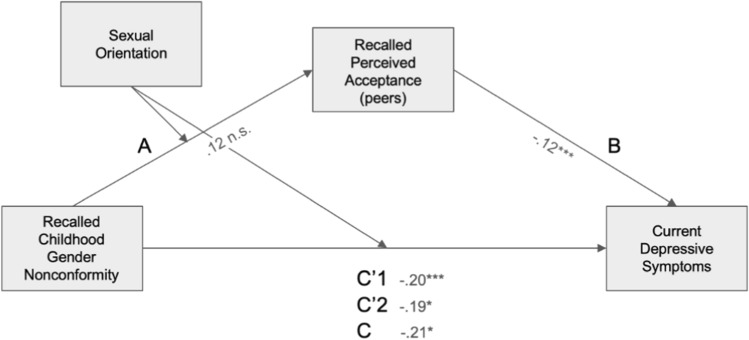
Mediated moderation model of the association between recalled childhood gender nonconformity and current depression scores mediated by recalled perceived peer acceptance levels and moderated by sexual orientation. Path coefficients are marked on the figure. Path statistics: A: *t*(789) = 1.61, *p* = .106; B: *t*(788) = − 3.36, *p* < .001; C: *t*(789) = − 5.42, *p* < .001; C’1 (gay): *t*(788) = − 3.95, *p* < .001; C’2 (heterosexual): *t*(788) = -2.66, *p* = .008. *significant at *p* < 0.05, ** significant at *p* < 0.01, *** significant at *p* < 0.001. Note that a higher score on the gender nonconformity scale corresponds to higher masculinity and therefore to lower childhood gender nonconformity

In the final model (Fig. 2), *R*^2^= 0.088, *F*(5, 788) = 15.2, *p* < 0.001. The direct association between CGN and depression scores for heterosexual men was significant (β =  − 0.20, *p* = 0.008, 95% CI [− 0.35, − 0.052]) as well as for gay men (β = − 0.18, *p* < 0.001, 95% CI [− 0.30, − 0.091]). The indirect association through recalled parental acceptance of CGN was not significant for heterosexual men (β = − 0.015, 95% CI [− 0.039, 0.004]), but significant for gay men (β = − 0.029, 95% CI [− 0.048, − 0.011]). However, the index of moderated mediation was nonsignificant, indicating that conditional indirect associations did not differ significantly between the two groups (β = 0.014, 95% CI [− 0.039, 0.008]).

#### Recalled Perceived Parental Acceptance and Social Anxiety Symptoms

**Fig. 3:**
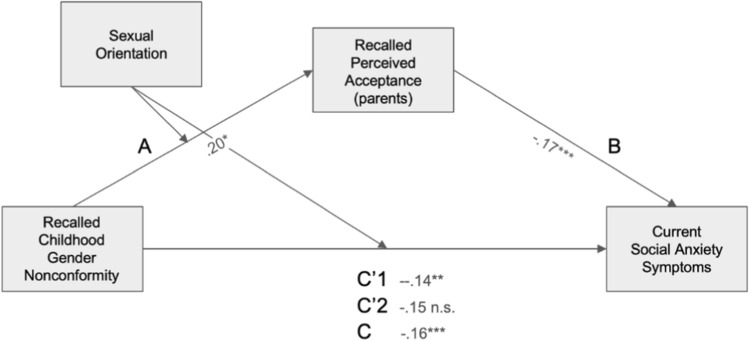
Mediated moderation model of the association between recalled childhood gender nonconformity and current social anxiety scores mediated by recalled perceived parental acceptance levels and moderated by sexual orientation. Path coefficients are marked on the figure. Path statistics: A: *t*(789) = 2.53, *p* = .012; B: *t*(788) = − 4.69, *p* < .001; C: *t*(789) = − 4.01, *p* < .001; C’1 (gay): *t*(788) = − 3.01, *p* = .003; C’2 (heterosexual): *t*(788) = − 1.90, *p* = .057. *significant at *p* < 0.05, ** significant at *p* < 0.01, *** significant at *p* < 0.001. Note that a higher score on the gender nonconformity scale corresponds to higher masculinity and therefore to lower childhood gender nonconformity

In the final model (Fig. 3), *R*^2^= 0.065, *F*(5, 788) = 11.0, *p* < 0.001. The direct association between CGN and social anxiety scores was not significant for heterosexual men (β = − 0.15, *p* = 0.057, 95% CI [− 0.30, − 0.005]) but significant for gay men (β = − 0.14, *p* = 0.003, 95% CI [− 0.23, − 0.047]). The indirect association through parental acceptance of CGN was significant for heterosexual men (β = − 0.032, 95% CI [− 0.063, − 0.007]), but not significant for gay men (β = − 0.015, 95% CI [− 0.033, 0.001]). As with depression scores, the index of moderated mediation was nonsignificant, indicating that conditional indirect associations did not differ significantly between the two groups (β = 0.017, 95% CI [− -0.011, 0.051]).

#### Recalled Perceived Peer Acceptance and Social Anxiety Symptoms

**Fig. 4:**
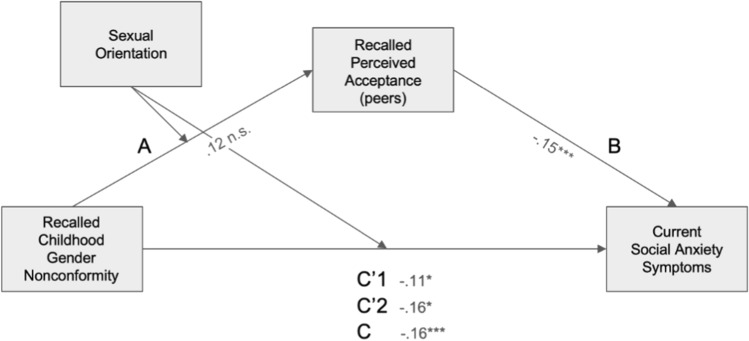
Mediated moderation model of the association between recalled childhood gender nonconformity and current social anxiety scores mediated by recalled perceived peer acceptance levels and moderated by sexual orientation. Path coefficients are marked on the figure. Path statistics: A: *t*(789) = 2.53, *p* = .012; B: *t*(788) = -4.29, *p* < .001; C: *t*(789) = − 4.01, *p* < .001; C’1 (gay): *t*(788) = − 2.46, *p* = .014; C’2 (heterosexual): *t*(788) = − 2.07, *p* = .038. *significant at *p* < 0.05, ** significant at *p* < 0.01, *** significant at *p* < 0.001. Note that a higher score on the gender nonconformity scale corresponds to higher masculinity and therefore to lower childhood gender nonconformity

In the final model (Fig. 4), *R*^2^= 0.06, *F*(5, 788) = 10.3, *p* < 0.001. The direct association between CGN and social anxiety scores was significant for heterosexual men (β = − 0.16, *p* = 0.038, 95% CI [− 0.31, − 0.009]) and significant for gay men (β = − 0.11, *p* = 0.014, 95% CI [ − 0.20, − 0.023). The indirect association through peers’ acceptance of CGN was not significant for heterosexual men (β = − 0.019, 95% CI [− 0.048, 0.005]) but was significant for gay men (β = − 0.038, 95% CI [− 0.059, − 0.018]). As before, the index of moderated mediation was nonsignificant, indicating that conditional indirect associations did not differ significantly between the two groups (β = 0.019, 95% CI [− 0.050, 0.010]).

### Mediation by Internalized Homophobia for Gay Men

#### Depressive Symptoms, Recalled Perceived Parental Acceptance

The lack of correlation between CGN and recalled perceived parental acceptance of CGN does not permit us to test for a mediating effect for this particular association.

#### Depressive Symptoms, Recalled Perceived Peer Acceptance

**Fig. 5:**
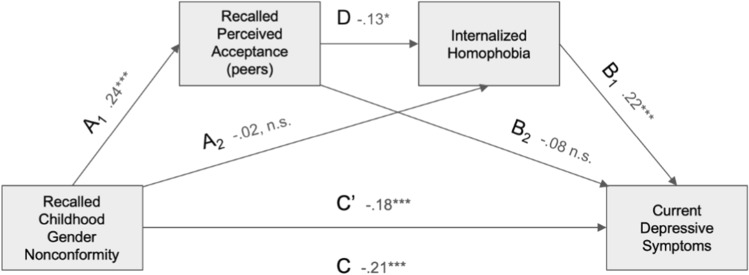
Model of the association between recalled childhood gender nonconformity and current depression scores in gay men, mediated by recalled perceived peers acceptance levels and internalized homophobia acting in serial. Coefficients and their *p*-values are shown in the figure. Path statistics: A_1_: *t*(449) = 5.43, *p* < .001; A_2_: *t*(448) = − 0.50, *p* = .617; B_1_: *t*(447) = 4.56, *p* < .001; B_2_: *t*(447) = − 1.55, *p* = .121; C: *t*(449) = − 4.38, *p* < .001; D: *t*(448) = -2.59, *p* = .010; C’: *t*(447) = − 3.72, *p* < .001. *significant at *p* < 0.05, ** significant at *p* < 0.01, *** significant at *p* < 0.001. Note that a higher score on the gender nonconformity scale corresponds to higher masculinity and therefore to lower childhood gender nonconformity

Figure 5 shows the model with coefficients and statistics for all indirect and direct paths. In the analyses of gay men (*n* = 452), with an additional mediator (internalized homophobia), for the total association model, *R*^2^ = 0.083, *F*(2, 449) = 20.3, *p* < 0.001, while for the indirect association model, *R*^2^= 0.13, *F*(4, 447) = 17.0, *p* < 0.001. As can be seen in the figure, the total association of CGN scores and reported depressive symptoms (model without mediators, corresponding to path C) was negative and significant (β = 0.21, *p* > 0.001, 95% CI [− 0.30, ULCI = −0.11]), and the direct association of CGN and reported depressive symptoms (i.e., after including peers’ recalled perceived acceptance and internalized homophobia) was also significant but smaller (path C’; β = 0.18, *p* > 0.001, 95% CI [− 0.27, − 0.083]), suggesting that mediation occurred.

Bootstrap analysis with recalled perceived levels of parental acceptance of CGN as a single mediator gave no significant indirect association (β = 0.018, 95% CI [− 0.045, 0.005]). However, with two mediators in serial, the indirect association was small but significant (β = − 0.007, 95% CI [− 0.013, − 0.002]). The indirect association from the bootstrap analysis with internalized homophobia as a single mediator was statistically insignificant (β = − 0.005, 95% CI [− 0.030, 0.017]).

#### Social Anxiety Symptoms, Recalled Perceived Parental Acceptance

The lack of correlation between CGN and recalled perceived parental acceptance of CGN does not permit us to test for a mediating effect.

#### Social Anxiety Symptoms, Recalled Perceived Peer Acceptance

**Fig. 6:**
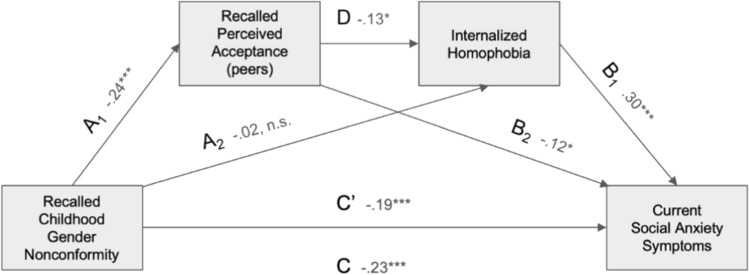
Model of the association between recalled childhood gender nonconformity and current social anxiety scores in gay men, mediated by recalled perceived peers acceptance levels and internalized homophobia acting in serial. Coefficients and their *p*-values are shown in the figure. Path statistics: A_1_: *t*(449) = 5.43, *p* < .001; A_2_: *t*(448) = − 0.50, *p* = .617; B_1_: *t*(447) = 6.39, *p* < .001; B_2_: *t*(447) = − 2.46, *p* = .014; C: *t*(449) = − 3.15, *p* = .002; D: *t*(448) = − 2.59, *p* = .010; C’: *t*(447) = − 2.26, *p* = .024. *significant at *p* < 0.05, ** significant at *p* < 0.01, *** significant at *p* < 0.001. Note that a higher score on the gender nonconformity scale corresponds to higher masculinity and therefore to lower childhood gender nonconformity

The model with coefficients and statistics for all indirect and direct paths is presented in Fig. 6. For gay men (*n* = 452), analyses performed with an additional mediator (internalized homophobia) entered into the model gave the following results: in the total association model, *R*^2^ = 0.42, *F*(2, 449) = 9.78, *p* < 0.001, and in the indirect association model, *R*^2^= 0.14, *F*(4, 447) = 18.3, *p* < 0.001. The total association between CGN and reported social anxiety symptoms (model without mediators, which corresponds to path C) was negative and significant (β = −0.15, *p* = 0.002, 95% CI [− 0.24, − 0.057]), and the direct association of gender conformity and social anxiety symptoms (i.e., after including mediators) was also significant but smaller (path C’; β = − 0.11, 95% CI − 0.20, − 0.014]). This suggests that mediation occurred.

Bootstrap analysis revealed that the indirect association with recalled perceived levels of peer acceptance of CGN as a single mediator was significant (β = − 0.007, 95% CI [− 0.041, 0.024]). The indirect association through two mediators in serial was slightly larger and also significant (β = 0.009, 95% CI [− 0.017, − 0.002]). However, the indirect association with internalized homophobia as a single mediator was statistically insignificant (β = 0.007, 95% CI [− 0.040, 0.023]).

## Discussion

The results support our first hypothesis. Gay men in our sample retrospectively reported being less gender conforming than did heterosexual men with a large effect size, as expected (Bailey & Zucker, 1995). However, our second hypothesis (that gay men would report higher levels of depressive and social anxiety symptoms) was not supported. Both groups of men reported similar scores for these symptoms—although the scores were slightly higher for gay men, the difference was not statistically significant. This is a surprising result. Moreover, in the case of social anxiety, both groups reported higher levels than those reported in nonclinical and clinical samples in the study investigating the psychometric properties of the tool used here (Kuncewicz et al., 2014). As is further discussed in the limitations section, it is possible that we obtained a rather unusual sample of heterosexual men.

Higher levels of recalled CGN were associated with less recalled perceived peer acceptance of CGN in gay men, and with less recalled perceived parental acceptance of CGN in heterosexual men. It is surprising that the CGN measure and the recalled perceived acceptance measures were not more strongly correlated and were not consistently correlated across groups. We assumed that men who were more gender nonconforming would have more instances of peer/parent reactions to reflect on, which could increase the likelihood that they would experience negative reactions. It is possible that we experienced some recruitment bias and only individuals more at peace with their sexuality (which could have been influenced by their childhood experiences) agreed to take part in the meeting (levels of internalized homophobia in our sample were overall rather low). It would be interesting to see what results would be observed if the study were conducted fully online.

Levels of retrospectively reported masculinity were significantly negatively correlated with reported adult depressive and social anxiety symptoms, and these relationships were significant for both gay and heterosexual men (although the correlations appear slightly smaller in the case of heterosexual men). This is consistent with previous studies showing an association between greater recalled childhood gender nonconformity and psychopathology independent of sexual orientation (Alanko et al., 2009; Roberts et al., 2013). However, inconsistently with our predictions, less gender nonconformity was not associated with lower levels of internalized homophobia, which is also in contrast with previous studies (Dragowski et al., 2011). However, greater perceived parental and peer acceptance of CGN scores were associated with lower levels of internalized homophobia, which is in line with previous reports (D'Augelli et al., 2008). As expected, internalized homophobia correlated positively with both depression and social anxiety symptom scores (cf. Dragowski et al., 2011; Feinstein et al., 2012).

The correlation analysis and the moderated mediation models suggest that recalled perceived parental acceptance of CGN played a role in psychological well-being of heterosexual men, while that of peers played a role for gay men. However, this needs to be considered with caution as tests verifying the statistical significance of moderated mediation found that neither the direct association between CGN and adult mental health nor the indirect association through either parents’ or peers’ recalled perceived acceptance levels was significantly moderated by sexual orientation. (This result could be due to the fact that the levels of mental health symptoms did not differ between the sexual orientation groups.)

We also expected that experiencing negative reactions to one’s gender-related behaviors in childhood would increase the risk of internalizing social stigma, and hence, internalized homophobia would subsequently mediate the relationship between CGN and psychosocial well-being in gay men. This expectation was partially supported by our analyses. In the case of depressive symptoms, we found that peers’ but not parents’ recalled perceived acceptance of CGN mediated this relationship, but only when both low acceptance and internalized homophobia were acting together. In the case of social anxiety, we also found that peers’ but not parents’ recalled perceived acceptance of CGN mediated this relationship, and here, both the acceptance on its own and acceptance and internalized homophobia acting in serial were significant mediators.

Thus, internalized homophobia was associated with a stronger relationship between a lack of recalled perceived peer acceptance of CGN and social anxiety symptoms. This is somewhat in line with a previous study that proposed a path model in which childhood gender nonconformity led to depressive and social anxiety symptoms through sexual-orientation-related experiences of discrimination in adulthood and internalized homophobia (Feinstein et al., 2012). However, in our models, internalized homophobia on its own was not a significant mediator of the relationship between recalled CGN and reported depressive and social anxiety symptoms.

It is interesting that our results suggest that peers play a more important role than parents—and more so for mediating the relationship between CGN and social anxiety than depressive symptoms in gay men. The former is in line with multiple reports about the important role that peer acceptance plays in the psychological well-being of children and adolescents (Newcomb et al., 1993) and fits with the notion of group socialization (Harris, 1995). The latter is interesting in the context of the emerging shift from the minority stress model toward the rejection sensitivity model, in which the feedback loop includes experiences of rejection and anxious expectations of rejection—a form of social anxiety (Downey & Feldman, 1996; Feinstein, 2020; Romero-Canyas et al., 2010). Peers have been previously reported to play important role in gender identity development (Kornienko et al., 2016), and self-perceived gender nonconformity has been found to undermine adjustment, in part by making adolescents feel more incompatible with their gender peer group (Menon, 2011). It should also be noted that in our sample parents were reported to be generally perceived as significantly more accepting than peers, which could have influenced the results (see Supplementary Material).

Another result worth paying attention to is the one suggesting that while peer acceptance is more important in the case of gay men, parental acceptance of CGN seemed more important in explaining the relationship between CGN and psychological well-being in heterosexual men. It could be speculated that because heterosexual men overall exhibited significantly more masculine behaviors in childhood, their answers reflected their overall relationship with parents more than just their acceptance of CGN in particular. It could also be that their levels of CGN were not high enough to affect their peer relationships—even if the responses to nonconformity were bad, they may not have happened frequently enough. Moreover, as previously mentioned, nonheterosexual individuals tend to report feeling “different” and being perceived as “different” already by the age of 8 (D'Augelli et al., 2008), and factors other than CGN that contribute to these perceptions could add to the perceived scrutiny of peers.

There may be other common causal factors that predict the traits examined in this study (e.g., genetic and environmental confounding). Future research should also consider the possibility of familial (genetic and/or environmental) confounding between mental health outcomes, sexual orientation, and psychological correlates such as internalized homophobia, since all these variables may be influenced by genetic and nongenetic factors. However, one study that used discordant monozygotic twins (which minimizes the influence of genetics on key phenotypic differences) suggests that some associations between minority stressors and mental health variables among LGB co-twins may be due to nonshared environmental factors (e.g., Timmins et al., 2018), whereas other studies (using both monozygotic and dizygotic twin pairs) suggest the involvement of common genetic components (Zietsch et al., 2012), while others are less able to separate sources of familial confounding (Donahue et al., 2017; Frisell et al., 2010). Other sources of confounding, such as personality factors (e.g., neuroticism), should also be studied (Bailey, 2021).

### Limitations

The present study had several strengths, including: a large sample from an Eastern European population which generated findings supportive of those found in Western European and North American samples, use of standardized measures appropriately translated, and sufficient power to detect mediation. However, there are some limitations worth noting.

The study focused on men and did not involve women or individuals of other sexual orientations, especially since the negative responses from others to female gender nonconformity are less clear in the literature. It is possible that some sampling biases occurred in the recruitment of heterosexual men. In fact, we experienced difficulty recruiting heterosexual men, perhaps as a result of fear or reluctance in taking part in a study advertised as concerning sexual orientation. Thus, it is possible that we obtained a rather unusual or more liberal sample of heterosexual Polish men. It also needs to be noted that the levels of CGN were higher in individuals who were included in the study than those in the initial sample, and more heterosexual than homosexual men did not answer the questions about perceived acceptance of their CGN (See Supplementary Material).

Our measure of recalled acceptance had only two items per scale and requires more psychometric testing for reliability and validity. The retrospective nature of part of our measures may introduce the risk of recall and other desirability biases. However, it should be noted that retrospective measures of childhood gender nonconformity are generally found to be highly reliable (Bailey & Zucker, 1995; Li et al., 2017; MacMullin et al., 2021; Rieger et al, 2008). It is possible that participants with lower levels of internalized homophobia were more likely to volunteer to take part in the study. Depressive and social anxiety symptoms were based on measurements from a symptom screening tool rather than a clinical diagnosis.

The cross-sectional and self-selected nature of the sample does not permit causal inferences to be made or for greater generalizability. There is always the possibility of reverse causation or other causal patterns (Bailey, 2020), given the cross-sectional and retrospective nature of the design. For example, poorer mental health may be associated with focusing on negative experiences when reporting acceptance levels from parents and peers, or this focus may be a result of rejection sensitivity (Feinstein, 2020). Competing mediation models should be tested in future studies.

### Conclusion

In conclusion, our results suggest that associations between recalled CGN and poorer mental health are at least partially mediated by recalled perceived parental and/or peer acceptance of gender nonconformity and, among gay men, internalized homophobia. The results could tentatively suggest that gender nonconformity is an early developmental risk factor for later mental health outcomes in gay men—one which is present before their sexual identity is fully formed. CGN may also be a risk factor for boys in general, irrespective of their sexual orientation. Factors such as others' acceptance of these behaviors may partially mediate this risk. In gay men, low levels of acceptance, especially by peers, may influence adult mental health, at least partially, by contributing to higher levels of internalized homophobia. Future studies should test the patterns reported here in longitudinal, population-based samples in order to allow us to make stronger inferences about their causal structure and improve their generalizability, as well as consider other possible mediators of the relationship between CGN and psychological well-being.

## Supplementary Information

Below is the link to the electronic supplementary material.

Supplementary file1 (DOCX 147 KB)
