# Supplementary material for: Childhood Gender Nonconformity and Recalled Perceived Parental and Peer Acceptance Thereof, Internalized Homophobia, and Psychological Well-Being Outcomes in Heterosexual and Gay Men from Poland
Source: Arch Sex Behav. 2022 Jun 2;51(4):2199–212. doi: 10.1007/s10508-021-02245-9 (PMC9192395; doi:10.1007/s10508-021-02245-9)
Supplement: Supplementary file 1 — Supplementary file1 (DOCX 147 KB) [file 10508_2021_2245_MOESM1_ESM.docx]

**Childhood Gender Nonconformity, Parental and Peer Acceptance, Internalized Homophobia, and Psychological Well-Being Outcomes in Heterosexual and Gay Men**

**SUPPLEMENTARY MATERIAL**

**Comparisons between the initial group and the final group.**

More heterosexual men (*n* = 88.20% of the sample) than gay men (*n* =63.12% of the sample) did not answer the questions regarding CGN acceptance (*z* = -3.44, *p* < .001).

In terms of the mental health of individuals who did and did not answer the question, there were no statistically significant differences, although, numerically, the scores indicated slightly less symptoms in those who did not (heterosexual men: depressive *M* = 0.86, *SD* = 0.87; anxiety *M* = 1.34, *SD* = 0.82; homosexual men: depressive *M* = 0.86, *SD* = 0.87; anxiety *M* = 1.20, *SD* = 0.88) than those who did answer these questions (heterosexual men: depressive *M* = 0.88, *SD* = 0.78; anxiety *M* = 1.34 , *SD* = 0.81; homosexual men: depressive *M* = 0.93, *SD* = 0.8 ; anxiety *M* = 1.41, *SD* = 0.87). This was in line with our expectations, as generally individuals who were more likely to answer questions regarding the recalled perceived acceptance of their CGN also exhibited higher levels of CGN (heterosexual men: *M* = 4.05, *SD* = 0.34 for those who answered the questions and *M* = 4.25, *SD* = 0.28 for those who did not, *t*(160) = -5.59, *p* < .001; gay men: *M* = 3.59, *SD* = 0.50 for those who answered the questions and *M* = 4.01, *SD* = 0.30 for those who did not, *t*(114 )= -9.34, *p* < .001).

**Associations between variables of interest.**

The correlations between all variables of interest for the whole group as well as for gay and heterosexual men separately are presented in Table s1.

*Whole sample*

When the whole sample was considered, CGN score correlated negatively and significantly with both depression (Pearson’s *r* = -.20, *p* < .001) and social anxiety symptom scores (Pearson’s *r* = -.16, *p* < .001). Recalled perceived parental acceptance of gender nonconformity was significantly negatively correlated with depression (Pearson’s *r* = -.14, *p* < .001) and social anxiety (Pearson’s *r* = -.16., *p* < .001). Recalled perceived peer acceptance of gender nonconformity significantly correlated negatively with depression (Pearson’s *r* = -.14, *p* < .001) and social anxiety (Pearson’s *r* = -.17, *p* < .001). CGN score significantly correlated positively with recalled perceived peer acceptance (Pearson’s *r* = .1, *p* < .001), and recalled perceived parental acceptance (Pearson’s *r* = .13, *p* < .001). The two mental health variables positively correlated with each other (Pearson’s *r* = .51, *p* < .001), as did the two measures of recalled perceived acceptance (Pearson’s *r* = .38, *p* < .001).

*Gay Men*

In gay men, CGN score correlated negatively and significantly with both depression (Pearson’s *r* = -.22, *p* < .001) and social anxiety symptom scores (Pearson’s *r* = -.16, *p* < .001). Recalled perceived parental acceptance of gender nonconformity was significantly correlated with depression (Pearson’s *r* = -.17, *p* < .001) and social anxiety (Pearson’s *r* = -.15., *p* < .001). Recalled perceived peer acceptance of gender nonconformity significantly correlated negatively with depression (Pearson’s *r* = -.14, *p* = .003) and social anxiety (Pearson’s *r* = -.18, *p* < .001). As expected, internalized homophobia also significantly correlated positively with depression (Pearson’s *r* = .25, *p* < .001) and social anxiety (Pearson’s *r* = -.32, *p* < .001), and negatively with parental (Pearson’s *r* = -.20, *p* < .001) and peer acceptance levels (Pearson’s *r* = -.13, *p* < .005). CGN score significantly correlated positively with recalled perceived peer acceptance (Pearson’s *r* = .25, *p* < .001), but not recalled perceived parental acceptance (Pearson’s *r* = .07, *p* = .119). CGN score was not correlated with internalized homophobia (Pearson’s *r* = -.07, *p* = .122). The two mental health variables positively correlated with each other (Pearson’s *r* = .50, *p* < .001), as did the two measures of recalled perceived acceptance (Pearson’s *r* = .38, *p* < .001).

*Heterosexual Men*

In heterosexual men, CGN score correlated significantly with depression scores (Pearson’s *r* = -.18, *p* < .001) and social anxiety (Pearson’s *r* = -.14 *p* = .010). CGN correlated significantly with recalled perceived parental (Pearson’s *r* = .12, *p* = .021) but not peer acceptance of CGN (Pearson’s *r* = .08, *p* = .136). Recalled perceived parental acceptance of gender nonconformity was not correlated with depression (Pearson’s *r* = -.09, *p* = .101) but was significantly negatively correlated with social anxiety (Pearson’s *r* = -.17, *p* = .002). Recalled perceived peer acceptance of gender nonconformity significantly correlated negatively with depression (Pearson’s *r* = -.15, *p* = .007) and social anxiety (Pearson’s *r* = -.17, *p* = .002). The two mental health variables positively correlated with each other (Pearson’s *r* = .53, *p* < .001), as did the two measures of recalled perceived acceptance (Pearson’s *r* = .40, *p* < .001)

**Sell Assessment of Sexual Orientation.**

The correlations between scale items for the entire group who completed the recruitment questionnaire are presented in Table s2. The correlations between scale items for the final sample included in our analyses are presented in Table s3.

The correlations appeared stronger in the final sample than in the sample who completed the recruitment questionnaire. As expected, in both cases, the correlations between the items on the attraction scale seem stronger than between the items on the attraction and the conduct scale. Sexual identity also appeared to be correlated very strongly with items on the sexual attraction scale, and less strongly with items on the sexual conduct scale. See Sell (1996) for details regarding the questionnaire.

**Recalled Perceived Acceptance Scale**

Below is the English translation of the Recalled Perceived Acceptance Scale.

Note: The scale was designed with a computerized approach in mind, and while the participants’ answers are scored on a scale between 0 and 100, the score was not displayed to the participants when they moved the slider. Participants had the option to indicate that questions do not apply to them as they never behaved or played this way or to indicate “I don’t remember.”

Some toys and ways of playing are traditionally considered more boyish
(e.g., cars, playing war), or more girly (e.g., dolls, playing house).

What reactions of the following people in your environment were you met with when you engaged in play that can be considered more girly?

Please mark your answer on the slider (referring to events before you were 12 years old). In order to select the middle answer (indifference) please click on the slider without moving it.

▢ The question does not apply to me, as I did not behave this way.

Peers

no acceptance <----------------------------------------------------------------> acceptance

indifference

▢ I don’t remember

Parents

no acceptance <----------------------------------------------------------------> acceptance

indifference

▢ I don’t remember

Some behaviors not associated with play are traditionally considered more boyish
(e.g., aggression), or more girly (e.g., crying).

What reactions of the following people in your environment were you met with, when you exhibited behaviors that can be considered more girly?

Please mark your answer on the slider (referring to events before you were 12 years old).

▢ The question does not apply to me, as I did not behave this way.

Peers

no acceptance <----------------------------------------------------------------> acceptance

indifference

▢ I don’t remember

Parents

no acceptance <----------------------------------------------------------------> acceptance

indifference

▢ I don’t remember

The score for the Recalled Perceived Peers’ Acceptance was obtained by calculating the mean answers on items regarding peers, and the score for the Recalled Perceived Parents’ Acceptance was obtained by calculating the mean answers on items regarding parents. Distributions of scores in our sample are presented in Figure s1.

Because of the observation that only peers’ but not parents’ acceptance was correlated with CGN in gay men, while only parents’ but not peers acceptance was correlated with CGN in heterosexual men, we decided to perform an additional analysis to formally check which people in one’s environment were perceived as less accepting in the two groups. To do so, we performed paired *t*-tests comparing the two measures in each of the groups. The results presented in Table s4 show that in our sample both gay and heterosexual men recalled perceiving their peers (for whom the mean score was below 50, and thus on the side of lack of acceptance) as less accepting that parents (for whom the mean score was above 50, indicating acceptance).

| **Table s1** |  |  |  |  |  |  |
| --- | --- | --- | --- | --- | --- | --- |
| *Pearson's correlations between the variables of interest for the whole group, as well as gay men, and heterosexual men separately* | | | | | | |
|  | CGN | Depression | Social anxiety | Parents' acceptance | Peers' acceptance | Internalized homophobia |
| Whole sample (*n* =794) | | | | | | |
| CGN |  | -.10*** | -.16*** | .12*** | .13*** | n.a. |
| Depression | -.20*** |  | .51*** | -.14*** | -.14*** | n.a. |
| Social anxiety | -.16*** | .51*** |  | -.16*** | -.17*** | n.a. |
| Parents' acceptance | .12*** | -.14*** | -.16*** |  | .38*** | n.a. |
| Peers' acceptance | .13*** | -.14*** | -.17*** | .38*** |  | n.a. |
| Gay men (*n*=452) | | | | | | |
| CGN |  | -.22*** | -.16*** | .07 n.s. | .25*** | -.07 n.s. |
| Depression | -.22*** |  | .50*** | -.17*** | -.14** | .25*** |
| Social anxiety | -.16*** | .50*** |  | -.15** | -.18*** | .32*** |
| Parents' acceptance | .07 n.s. | -.17*** | -.15** |  | .38*** | -.20*** |
| Peers' acceptance | .25*** | -.14** | -.18*** | .38*** |  | -.13** |
| Internalized homophobia | -.07 n.s. | .25*** | .32*** | -.20*** | -.13** |  |
| Heterosexual men (*n*=342) | | | | | | |
| CGN |  | -.18** | -.14* | .12* | .08 n.s. | n.a. |
| Depression | -.18** |  | .53*** | -.09 n.s. | -.15** | n.a. |
| Social anxiety | -.14* | .53*** |  | -.17** | -.17** | n.a. |
| Parents' acceptance | .12* | -.09 n.s. | -.17** |  | .40*** | n.a. |
| Peers' acceptance | .08 n.s. | -.15** | -.17** | .40*** |  | n.a. |
| *Note*. *** Correlation is significant at the < .001 level (2-tailed). ** Correlation is significant at the .01 level (2-tailed). ** Correlation is significant at the .01 level (2-tailed). * Correlation is significant at the .05 level (2-tailed). * Correlation is significant at the .05 level (2-tailed).  n.s., No significant correlation. n.a., Not applicable. | | | | | | |

| **Table s2** | | | | | | | | | | | | |
| --- | --- | --- | --- | --- | --- | --- | --- | --- | --- | --- | --- | --- |
| *Pearson’s correlations between the items of the Sell Assessment of Sexual Orientation Scale in all individuals who completed the recruitment questionnaire (n=3515).* | | | | | | | | | | | | |
|  | 1 | 2 | 3 | 4 | 5 | 6 | 7 | 8 | 9 | 10 | 11 | 12 |
| I Sexual Attractions | | | | | | | | | | | | |
| 1. Number of men one was attracted to |  | .85 | .85 | -.58 | -.68 | -.68 | .67 | .59 | -.49 | -.59 | .81 | -.76 |
| 2. Frequency of attraction to men | .85 |  | .89 | -.64 | -.72 | -.73 | .63 | .69 | -.52 | -.62 | .84 | -.78 |
| 3. Intensity of attraction to men | .85 | .89 |  | -.66 | -.74 | -.73 | .65 | .69 | -.53 | -.64 | .88 | -.82 |
| 4. Number of women one was attracted to | -.58 | -.64 | -.66 |  | .84 | .83 | -.51 | -.56 | .60 | .60 | -.70 | .75 |
| 5. Frequency of attraction to women | -.68 | -.72 | -.74 | .84 |  | .91 | -.58 | -.62 | .62 | .75 | -.84 | .81 |
| 6. Intensity of attraction to women | -.68 | -.73 | -.73 | .83 | .91 |  | -.57 | -.62 | .63 | .73 | -.86 | .84 |
| III Sexual Identity | | | | | | | | | | | | |
| 11. Extent of identification as homosexual | .81 | .84 | .88 | -.78 | -.84 | -.86 | .65 | .69 | -.60 | -.70 |  | -.89 |
| 12. Extent of identification as heterosexual | -.76 | -.78 | -.82 | .75 | .81 | .84 | -.60 | -.64 | .57 | .67 | -.89 |  |

*Note.* All correlations are significant at *p* < .001 level (2-tailed).

| **Table s3** | | | | | | | | | | | | | |
| --- | --- | --- | --- | --- | --- | --- | --- | --- | --- | --- | --- | --- | --- |
| *Pearson’s correlations between the items of the Sell Assessment of Sexual Orientation Scale in the sample analysed in the study (n=794).* | | | | | | | | | | | | | |
|  | 1 | 2 | 3 | 4 | 5 | 6 | 7 | 8 | 9 | 10 | 11 | 12 |  |
| I Sexual Attractions | | | | | | | | | | | | | |
| 1. Number of men one was attracted to |  | .89 | .90 | -.81 | -.83 | -.86 | .74 | .70 | -.61 | -.71 | .90 | -.90 |  |
| 2. Frequency of attraction to men | .89 |  | .92 | -.81 | -.83 | -.86 | .70 | .76 | -.61 | -.71 | .91 | -.90 |  |
| 3. Intensity of attraction to men | .90 | .92 |  | -.86 | -.88 | -.90 | .72 | .76 | -.64 | -.75 | .95 | -.95 |  |
| 4. Number of women one was attracted to | -.81 | -.81 | -.86 |  | .88 | .88 | -.65 | -.69 | .67 | .69 | -.90 | .89 |  |
| 5. Frequency of attraction to women | -.83 | -.83 | -.88 | .88 |  | .94 | -.67 | -.70 | .67 | .78 | -.91 | .91 |  |
| 6. Intensity of attraction to women | -.86 | -.86 | -.90 | .88 | .94 |  | -.69 | -.73 | .70 | .79 | -.94 | .94 |  |
| III Sexual Identity | | | | | | | | | | | | | |
| 11. Extent of identification as homosexual | .90 | .91 | .95 | -.90 | -.91 | -.94 | .72 | .76 | -.68 | -.77 |  | -.99 |  |
| 12. Extent of identification as heterosexual | -.90 | -.90 | -.95 | .89 | .91 | .94 | -.71 | -.75 | .67 | .77 | -.99 |  |  |

*Note.* All correlations are significant at *p* < .001 level (2-tailed).

| **Table s4** |  | |  | |  |  |  | |
| --- | --- | --- | --- | --- | --- | --- | --- | --- |
| *Paired comparisons of acceptance scores for parents and peers within the two groups.* | | | | | | | | |
| Comparison | | Mean | SD | *t* | | df | Significance (2-tailed) | Cohen's *d* |
| Heterosexual men | | | | | | | | |
| Parents' Acceptance - Peers' Acceptance | | 25 | 24.8 | 18.3 | | 341 | *p* < .001 | 24.8 |
| Homosexual men | | | | | | | | |
| Parents' Acceptance - Peers' Acceptance | | 17 | 24.9 | 14.5 | | 451 | *p* < .001 | 25 |


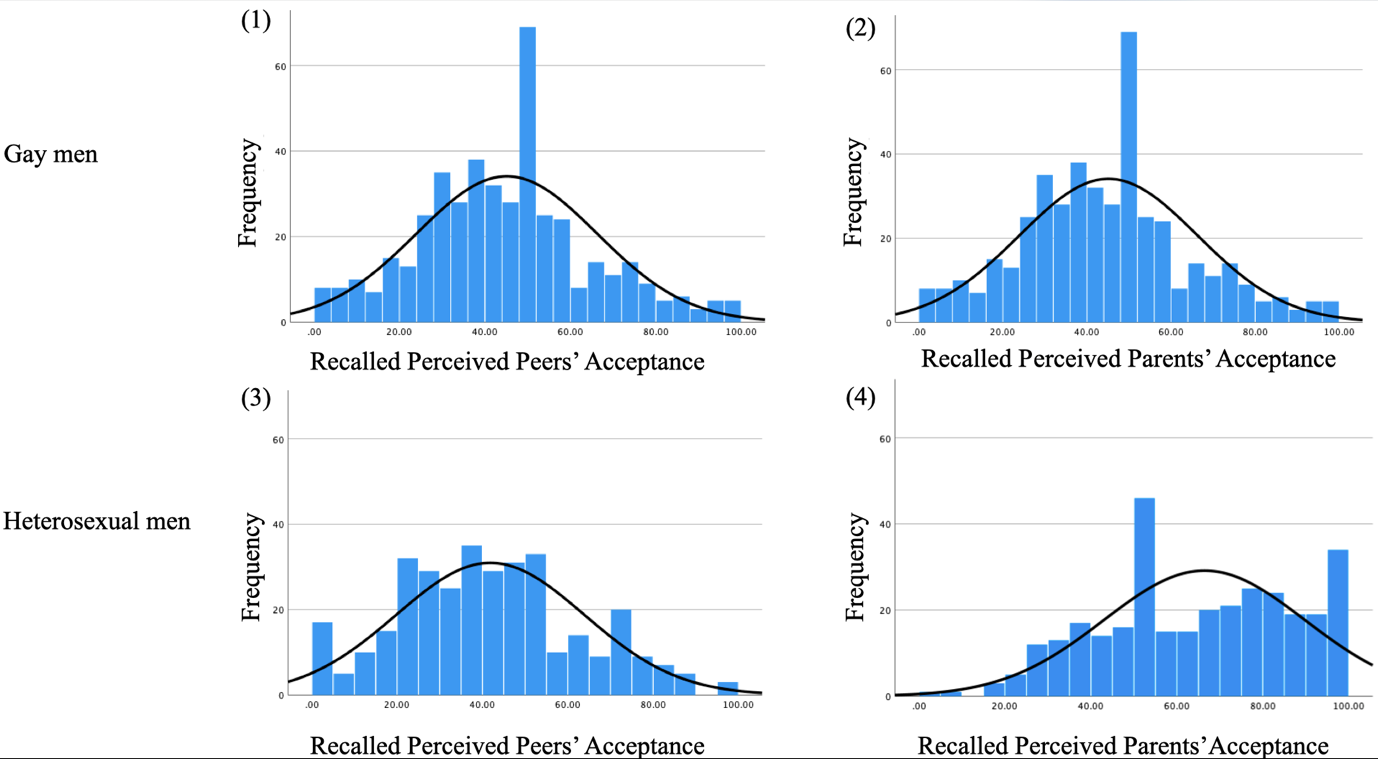
Figure s1

Histograms representing the distribution of scores on the acceptance scales by sexual orientation: (1) distribution of gay men scores on the Recalled Perceived Peers’ Acceptance scale, (2) distribution of gay men scores on the Recalled Perceived Parents’ Acceptance scale, (3) distribution of heterosexual men scores on the Recalled Perceived Peers’ Acceptance scale, (4) distribution of heterosexual men scores on the Recalled Perceived Parents’ Acceptance scale. Normal curve is shown on each graph. The scores range from 0 to 100, where 0 indicates no acceptance, 50 indicates indifference, and 100 indicates full acceptance.
